# Warfarin: The End or the End of One Size Fits All Therapy?

**DOI:** 10.3390/jpm8030022

**Published:** 2018-06-28

**Authors:** Munir Pirmohamed

**Affiliations:** Department of Molecular and Clinical Pharmacology, Wolfson Centre for Personalised Medicine, University of Liverpool, Liverpool L69 3GL, UK; munirp@liverpool.ac.uk; Tel: +44-151-794-5549

**Keywords:** warfarin, vitamin K antagonists, direct oral anticoagulants, dabigatran, rivaroxaban, apixaban, edoxaban, pharmacogenomics, personalized medicine, therapeutic drug monitoring

## Abstract

Oral anticoagulants are required for both treatment and prophylaxis in many different diseases. Clinicians and patients now have a choice of oral anticoagulants, including the vitamin K antagonists (of which warfarin is the most widely used and is used as the exemplar in this paper), and direct oral anticoagulants (DOACs: dabigatran, apixaban, rivaroxaban, and edoxaban). This paper explores the recent advances and controversies in oral anticoagulation. While some commentators may favour a complete switchover to DOACs, this paper argues that warfarin still has a place in therapy, and a stratified approach that enables the correct choice of both drug and dose would improve both patient outcomes and affordability.

## 1. Introduction

Warfarin is more than 60 years old. With the advent of direct oral anticoagulants (DOACs), some commentators have suggested that the end for warfarin is nigh [1]. However, it may be premature to write an obituary for warfarin, given its widespread use worldwide and the inability to use warfarin in some patient groups, such as in children, in patients with renal impairment, and in patients with heart valves. Furthermore, given the cost of DOACs, there may still be a place in clinical practice for a stratified approach to anticoagulation. This article examines the history of warfarin use, and in particular, the role of pharmacogenetics, and looking into the future, what still needs to be done to improve the benefit–risk ratio for all oral anticoagulants.

## 2. Warfarin Pharmacology and Pharmacogenetics

Warfarin is a vitamin K antagonist; it inhibits vitamin K epoxide reductase complex I (VKORC1), preventing the formation of activated vitamin K-dependent coagulation factors II, VII, IX, and X [2]. The gene *VKORC1* is polymorphically expressed, which leads to variable expression and activity of the enzyme; for example, the *−1639A* variant at rs9923231 leads to reduced mRNA levels [3]. Warfarin is administered as a racemate, with the more potent enantiomer *S*-wafarin being metabolised by the P450 isoform CYP2C9 [2]. *CYP2C9* genetic variants show reduced activity; for example, CYP2C9*3 is associated with a 90% reduction in catalytic activity of the enzyme [4]. It is interesting to note that the frequencies of both *VKORC1* and *CYP2C9* genetic polymorphisms vary with ethnicity, which has an impact on dose requirement worldwide [5,6]. It is widely acknowledged that African patients generally require higher doses than Caucasians, while Chinese patients require lower doses.

There is no doubt that warfarin is an effective drug for the treatment of venous thromboembolism and for prophylaxis against strokes in patients with atrial fibrillation [7]. However, warfarin is also associated with bleeding, estimated to be about 7.2 events per 100 patient years [8]. Indeed, warfarin is amongst the top three drugs responsible for adverse drug reaction related hospital admissions [9]. The major issue with warfarin is the inability to predict inter-individual variability in daily dose requirements. Some patients require 0.5 mg per day to maintain therapeutic anticoagulation, for example—an International Normalised Ratio (INR) between 2 and 3 in the treatment of atrial fibrillation—while others may require 20 mg per day—a variability of more than 40-fold. We have known for many decades that this variability is partly dependent on clinical factors, such as age, body-mass index (BMI), drug–drug interactions, and concomitant diseases [2]. However, apart from age, none of these clinical predictors have routinely been utilised in dosing regimens.

In order to improve the predictability of daily warfarin dose requirements, many groups worldwide have evaluated the role of genetic and clinical factors. These studies have consistently shown that genetic polymorphisms in *CYP2C9* and *VKORC1* account for a greater degree of variance in daily dose requirement compared with clinical factors, such as age, BMI, and interactions with drugs such as amiodarone [10,11]. Indeed, the association of warfarin dose requirement with genetic factors is one of the most highly replicated genotype–phenotype associations. Another genetic factor associated with the warfarin dose requirement is the *CYP4F2* genetic variant at rs2108622 (*V433M*) [12], the effect being due to differential vitamin K hydroxylation [13]. However, the overall effect size of *CYP4F2* is much lower than that of *CYP2C9* and *VKORC1*, accounting for about 1% of the variability in the warfarin dose requirement [14].

One of the most important developments in warfarin pharmacogenetics was the formation of the International Warfarin Pharmacogenetics Consortium (IWPC) that brought together 21 research groups from nine countries and four continents, with analysable data from about 5000 patients [15]. The IWPC developed clinical and genetic warfarin dosing algorithms. The genetic dosing algorithm was superior to the clinical dosing algorithm, with the greatest benefit being observed in about half of the patients who required either less than 21 mg of warfarin per week or more than 49 mg per week.

An important next step in the demonstration of the utility of genotype-guided dosing for warfarin was to demonstrate whether the genetic algorithm was superior to either standard care or a clinical algorithm. A number of clinical trials had already been conducted in this area with variable designs and disparate sample sizes, with inconclusive findings [16]. Because of this, two larger randomized controlled trials (RCTs) were undertaken, one in the United States (Clarification of Optimal Anticoagulation through Genetics; COAG) [17] and another in the European Union (EU Pharmacogenetics of Anticoagulant Therapy; EU-PACT) [18]. There were numerous differences in the design of the two trials [16], with COAG comparing a clinical algorithm to a genetic algorithm, while EU-PACT compared standard care in two European countries (the United Kingdom (UK) and Sweden) to the genetic algorithm. Unfortunately, but perhaps not surprisingly, given the differences in trial design, the results of the two trials were starkly different, with EU-PACT showing the superiority of the genetic algorithm over the comparator arm [19], while in COAG there was no difference between the test and control arms [20]. This led to confusion and criticisms of both trials and the conclusion that pharmacogenetic dosing of warfarin was not clinically useful [21,22]. Perhaps, this is consistent with the recent paper in *Science*, which highlighted that negative stories are more likely to get traction when compared with positive news [23]. Interestingly, following the publication of the trials, it was also suggested that dosing should be based on a clinical algorithm, rather than the standard dosing currently used in clinical care, despite the fact that there has been no RCT that has compared a clinical algorithm to standard of care. This is perhaps another example of genetic exceptionalism, where a lower burden of proof is considered acceptable for non-genetic interventions when compared with genetic testing. In retrospect, it would have been better to conduct three-armed trials, where genotype-guided dosing was compared simultaneously with both a clinical algorithm and standard dosing.

The reasons for the different outcomes in the two trials have been widely debated and included differences in algorithms, differences in the comparator arms, and greater ethnic heterogeneity in COAG compared with EU-PACT [16]. The design of any algorithm is, of course, crucial, and it must take into account ethnic heterogeneity (including the relevant ethnic-specific single nucleotide polymorphisms (SNPs)) and the known pharmacology of warfarin, including its long half-life. It is also fair to state that no algorithm is perfect, because not all factors that determine warfarin dosing have been identified [10]. The algorithms also predict extreme doses less efficiently (i.e., low or high daily warfarin doses) [15,24]. Algorithms designed for African-American, Indian, and Chinese patients have been developed but have not been tested in clinical trials [25,26,27].

More recently, the Genetics Informatics Trial (GIFT) has been published [28], conducted in 1650 randomised patients. This used a composite primary outcome measure comprising major bleeding, INR of four or greater, venous thromboembolism, or death. The trial found that 10.8% of the genotype-guided group met at least one of the endpoints compared with 14.7% in the clinically guided warfarin group, a 27% improvement in favour of genotype-guided dosing. The results are consistent with EU-PACT [19], but GIFT has the advantage of having included clinical outcomes as part of the primary endpoint. Indeed, EU-PACT and COAG were both criticised for having used percentage time in therapeutic range (%TTR), a surrogate measure, as the primary outcome measure [22]. Of course, a trial that used only clinical events (thromboembolic or bleeding) as the primary outcome would be preferable but would have required a sample size close to 20,000 patients. It is also important to note that an improvement in %TTR greater than 10% can lead to a 20% improvement in clinical outcomes [29].

From the EU-PACT trial data, it has been shown that genotyping prior to warfarin prescription would be cost effective in both the UK and Sweden [30]. Data on cost-effectiveness from other healthcare systems is awaited [31]. The 2017 Clinical Pharmacology Implementation Consortium (CPIC) guideline on warfarin provides detailed guidance on dosing in patients with variant alleles, including race-specific recommendations [32]. The Dutch Pharmacogenetic Working Group has also developed guidelines for the dosing of vitamin K antagonists, and there is a high overall rate of concordance between the guidelines produced by the Dutch Group and the CPIC [33].

## 3. Direct Oral Anticoagulants

Oral anticoagulants acting via the vitamin K-dependent pathway were the only choices available for clinicians until recently. The introduction of direct acting oral anticoagulants (DOACs) has provided greater choice for both clinicians and patients. These drugs act by inhibiting either thrombin (dabigatran) or factor Xa (apixaban, rivaroxaban, edoxaban). Large RCTs in atrial fibrillation and venous thromboembolism have shown that DOACs are either non-inferior or superior to warfarin, with a reduced risk of intracranial haemorrhage but with a possibly increased risk of gastro-intestinal bleeding [34].

DOACs have been marketed on the basis that “one-dose-fits-all” and that no monitoring is required. There has been rapid uptake of these drugs particularly in western countries, while the use of warfarin has declined [1]. This may well herald the beginning of the end for warfarin, but I feel this is premature for several reasons.

First, although the new DOACs have been shown to be cost-effective [35], there are concerns about the cost outlay given the large population that needs to be treated. It has been estimated that there are 8.8 million people with atrial fibrillation in the European Union, and this will double to 17 million in 2060 [36]. In the UK, it has been estimated that expenditure on DOACs may top £1 billion per year by 2020, about 5% of the overall spend on drugs in the National Health Services (NHS) [37]. One possible method to improve affordability and ensure that all patients have access to oral anticoagulation would be to stratify treatment according to genotypes for *CYP2C9* and *VKORC1*. The evidence for this comes from analyses of the warfarin arm in the edoxaban ENGAGE AF-TIMI 48 trial [38]. This showed that warfarin increased the risk of bleeding in those patients who carried variant alleles for *CYP2C9* and/or *VKORC1*, classified as sensitive or highly sensitive responders, when compared with normal responders, who represented 62% of the population. A more recent analysis of the Hokusai-venous thromboembolism trial [39] has replicated this finding, showing that sensitive and highly sensitive responders spent more time over-anticoagulated with warfarin and had a higher bleeding risk compared with normal responders (who represented 63% of the population). Thus, it may be possible to personalise the use of oral anticoagulants in the future so that patients with the low-risk genotypes (i.e., normal responders, at least 60%) would get warfarin, while those classified as sensitive or highly sensitive would get DOACs. This would lead to significant savings in expenditure [37] without any compromise in clinical outcomes.

An important issue to consider for taking forward the stratification approach is whether it is possible to implement warfarin genotyping in a clinical setting. Following the EU-PACT trial [19], and despite the conflicting results with COAG [20], we have undertaken an implementation study. The premise behind the study was to determine whether staff running anticoagulant clinics (predominantly qualified nurses) could modify the current clinical pathway so that genotyping, and subsequent genotype-guided dosing, could be incorporated. This required an improvement in the point-of-care genotyping assay. In EU-PACT, the point-of-care genotyping platform was able to provide results on three alleles (CYP2C9*2, CYP2C9*3, and *VKORC1*) in 2 h [19]. For the implementation study, the platform was modified to provide results within 45 min. The results of the implementation study (unpublished) were equivalent to those of the EU-PACT RCT, demonstrating that long and well-established clinical pathways could be modified using new technologies.

Second, an unintended consequence of the “no or minimal monitoring” strategy adopted for DOACs may be poor adherence. Our recent data suggest that adherence to DOACs was significantly worse when compared with warfarin [37]. Although INR monitoring is disliked by both patients and clinicians, it does act as a positive reinforcement for patients to continue taking warfarin. It is important to note that there has also been criticism of the “one-dose-fits all” strategy for DOACs, with some patients having been shown to be under-dosed, while others may be over-dosed [40]. Pharmacogenomic studies have identified some associations with plasma concentrations of these drugs, but none of the genetic variants are likely to be useful in improving the clinical use of these drugs [41,42]. Instead, it has been argued that plasma therapeutic drug monitoring should be utilised [43], especially in high-risk patients to individualise dose. High-risk groups could include patients at high risk of bleeding, those on drugs likely to interact with DOACs, and patients with borderline renal impairment, to name a few. Clearly, this would lead to a loss of the marketing advantage for DOACs, increase cost and inconvenience for patients, and may thus face an uphill battle for implementation.

Third, there are some patients where DOACs are not used because of contraindications, no marketing authorization, or unaffordability. In these situations (outlined below), warfarin remains the only alternative.

(a) ***Patients with renal impairment***: all the drug labels for DOACs have criteria, which either recommend a dose reduction or absolutely contraindicate the use of the DOAC [44]. While it may be relatively easy to avoid the use of DOACs in patients with severe forms of renal impairment, a group that may be at particular risk are patients with incipient renal impairment, where there may be asymptomatic and slow decline in renal function with age or an acute decline in an elderly patient because of a concomitant urinary tract infection. This is compounded by the fact that monitoring of renal function in patients on DOACs is poorly performed.
(b) ***Patients on interacting drugs***: Although DOACs are less likely to be involved in drug–drug interactions than warfarin, they are not immune from them. For patients on certain medications—for example, itraconazole—the use of apixaban is not recommended. A recent database study from Taiwan showed that concomitant use of drugs, such as amiodarone, fluconazole, rifampicin, and phenytoin, increased the risk of major bleeding when compared with the use of DOACs alone [45]. There is no simple biomarker that can be used to individualise dosing with DOACs, unlike warfarin, where INR monitoring provides the opportunity to change dosage to maintain the INR within a therapeutic range.
(c) ***Use in children***: DOACs are currently not licensed for use in children, but there are paediatric investigation plans in place [46]. Thus, for the time being, warfarin (or other vitamin K antagonists) remain the only alternatives. There have been numerous studies in children investigating the effects of genetic polymorphisms on warfarin dosing [47,48,49,50], but no algorithm has been tested in clinical trials.
(d) ***Mechanical heart valves****:* DOACs are currently contraindicated in patients with mechanical heart valves. In the RE-ALIGN trial, after enrolment of 252 patients, an increased risk of bleeding and thrombosis was seen in patients on dabigatran, compared with warfarin, which resulted in premature discontinuation of the trial [51].

Fourth, because of the cost of DOACs, their uptake in developing countries has been low, and thus, warfarin (or other vitamin K antagonists) remains the main choice. Unfortunately, the genetic and clinical factors affecting warfarin dose variation have been poorly studied in developing countries, and, even in developed countries, in minority groups compared with Caucasian populations [6]. The frequencies of genetic variants in *CYP2C9* and *VKORC1* vary with ethnicity. Thus, in Chinese patents, CYP2C9*2 is less important than in Caucasians [52]. In African-Americans, both CYP2C9*2 and CYP2C9*3 have a low prevalence, with other variant alleles (*8, *11) being more important [6]. The importance of this was highlighted by the COAG trial [20], where genotype-guided dosing actually fared worse than clinical dosing in African-American patients. In Africa, where access to medicines and services is limited, warfarin remains the obvious choice for oral anticoagulation. However, dosing regimens are largely empirical and not evidence-based, which—coupled with the lack of infrastructure—leads to poor quality of anticoagulation. For example, in South Africa, a recent study showed that only 28% of patients achieved a therapeutic INR [53]. The importance of achieving better anticoagulation control in developing countries is shown by a study in South Africa, which demonstrated that haemorrhage was the fourth most common cause of hospital admission, with warfarin accounting for 68% of the bleeds [54]. We have recently embarked on a large programme of work in Uganda and South Africa (War-PATH: WARfarin anticoagulation in PATients in Sub-SaHaran Africa; http://warpath.info/), the aim of which is to identify the clinical and genetic factors determining variation in daily warfarin dose requirements and thereby, develop better clinical and genetic dosing algorithms to improve anticoagulation quality.

## 4. Conclusions

**Figure 1:**
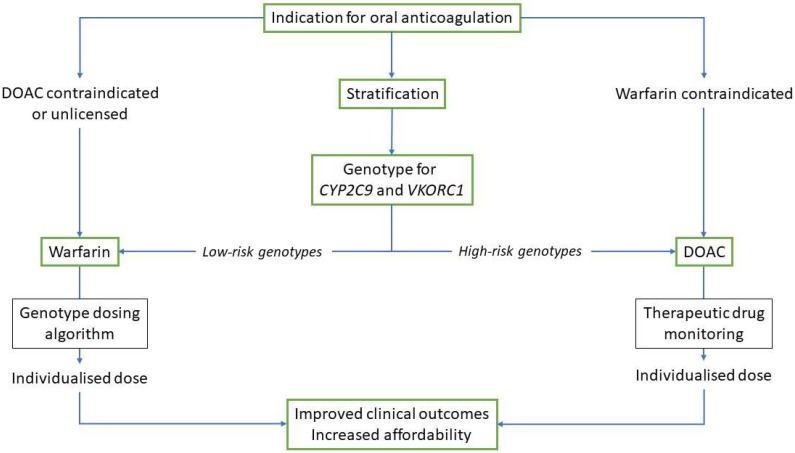
Clinical pathways for stratification in the use of oral anticoagulants. Use of either warfarin or a direct oral anticoagulant (DOAC) would require individualisation of the dose to improve time in therapeutic range and optimisation of anticoagulation, resulting in improved clinical outcomes. This would also result in improved affordability.

It has been estimated that the global anticoagulants market will be worth close to $30 billion by 2021 (https://www.businesswire.com/news/home/20170301005087/en/Increase-Lifestyle-Diseases-Boost-Global-Anticoagulant-Market). For both clinicians and patients, it is important to have a choice of drugs to use for either treatment or prophylaxis, and the availability of DOACs has certainly provided that choice. However, that does not mean that older drugs have no place in the therapeutic armamentarium, as I have pointed out in the case of warfarin. Undoubtedly, we can continue to improve the benefit–risk ratio of all oral anticoagulants that are available, and a stratified approach to the choice of drug, and the precise dose of that drug (Figure 1), may be an option that not only maximises the positive clinical outcomes but also improves affordability and access. Whether this would be a cost-effective approach would need further study; however, it is clear that the cost-effectiveness of DOACs is reduced or nullified when the quality of anticoagulation with warfarin improves [55], which is likely to be the consequence of a stratified approach.
